# Adrenal Myelolipoma Masquerading as an Adrenal Malignancy

**DOI:** 10.1155/2022/4044602

**Published:** 2022-01-17

**Authors:** Davida A. Robinson, Margaret Kemeny, Juliana E. Muchinyi, Madiha Yasin, Nilda I. Montes, Sandeep Tuli, Radhika Jaiswal, Amanda Carter, Michal-Ann Derezil, Hanna Jang, David Reich

**Affiliations:** ^1^Icahn School of Medicine at Mount Sinai, Queens Hospital Center, Department of Surgery, 82-68 164th Street, New York, NY 11432, USA; ^2^Zucker School of Medicine at Hofstra and Northwell, Northwell Health-North Shore University Hospital, Department of Medicine, Division of Endocrinology, 500 Hofstra University Hempstead, New York, NY 11549, USA; ^3^Ichahn School of Medicine at Mount Sinai- Queens Hospital Center, Department of Radiology, 82-68 164th Street, New York, NewYork 11432, USA; ^4^Ichahn School of Medicine at Mount Sinai- Queens Hospital Center, Department of Medicine, Division of Endocrinology, 82-68 164th Street, New York, NewYork 11432, USA

## Abstract

An adrenal myelolipoma presenting with suspicious features may pose a diagnostic challenge to surgeons and endocrinologists. In this case report of an adult patient with undiagnosed congenital adrenal hyperplasia presenting with bilateral adrenal masses, we review his radiographic and clinical findings which were highly suspicious for adrenal malignancy. Features of adrenal myelolipoma that may resemble malignant lesions are reviewed. This case report highlights important features of adrenal myelolipoma that the surgeon and endocrinologist should be aware of. The importance of avoiding overtreating adrenal myelolipomas presenting as tumors of uncertain malignant potential is crucial.

## 1. Introduction

Adrenal myelolipoma (AML) is most commonly asymptomatic and diagnosed incidentally on imaging studies obtained for other indications unrelated to the adrenal mass [1]. The majority of adrenal incidentalomas are benign adrenocortical adenomas, with only a 1.9% incidence of cancer [1]. Myelolipomas generally are benign tumors occurring in the adrenal gland and rarely in extra-adrenal locations that are characterized by mature adipose tissue intermixed with hematopoietic elements [2–4]. Likewise, AMLs are most commonly incidental findings, accounting for up to 15% of adrenal incidentalomas [2, 5, 6]. The exact pathophysiology and histogenesis of AML are not well understood; however, a possible mechanism involves adrenocortical metaplasia of capillary reticuloendothelial cells in response to pathologic stimuli (e.g., trauma and infection) or long-term ACTH stimulation, which in turn results in cellular differentiation of mesenchymal stem cells into myeloid and adipose tissues [5].

AMLs have characteristic radiographic and microscopic features, yet diagnosis of AML is not always straightforward and may present diagnostic challenges to the clinician. We present a case of a large AML masquerading as an invasive adrenal malignancy in a patient with previously undiagnosed (and therefore untreated) congenital adrenal hyperplasia (CAH) due to 21-hydroxylase deficiency (21-OHD) diagnosed in adulthood.

## 2. Case Presentation

**Figure 1:**
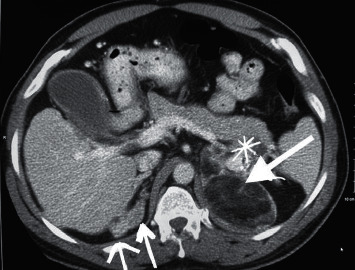
A contrast-enhanced computed tomography (CT) image demonstrating two nodules in the right adrenal gland (small arrows) and a large, left adrenal mass with calcifications (large arrow) and suspected invasion of the left adrenal mass into the tail of the pancreas (asterisk).

**Figure 2:**
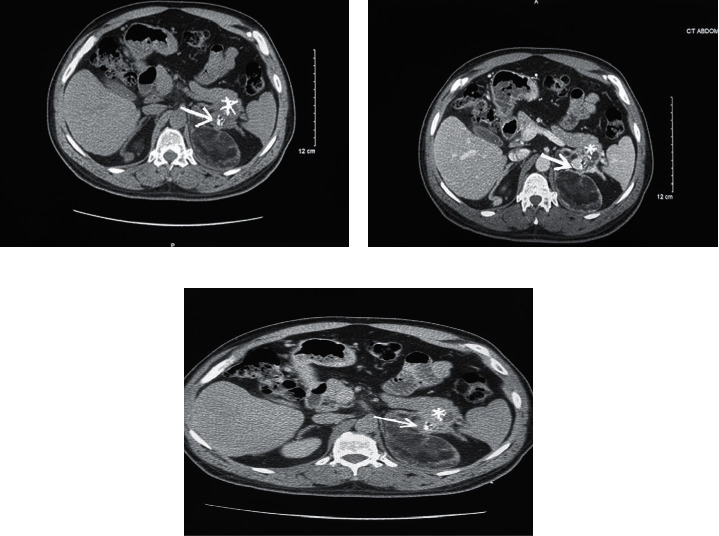
A multiphase CT with noncontrast (a), contrast (b), and delayed (c) images demonstrating the enhancing solid component of the left adrenal mass (arrow) and the region of questioned pancreatic invasion (asterisk).

**Figure 3:**
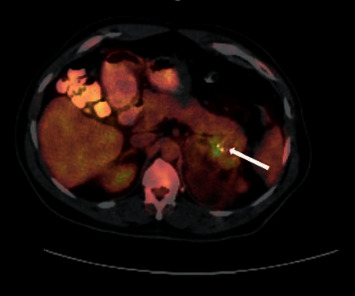
A positron emission tomography (PET) scan demonstrating metabolic uptake with focality localizing to the enhancing solid component of the left adrenal mass seen on the multiphase CT study.

A 48-year-old male presented to the surgical oncology clinic for follow-up of bilateral adrenal masses detected incidentally on computed tomography (CT) scan of the abdomen and pelvis obtained for unrelated indications. The patient had no significant medical history. On exam, he was noted to have short stature at 139.7 cm but was otherwise normally developed. Blood pressure was 115/76 mmHg and pulse was 76 beats per minute. No abdominal masses, lymphadenopathy, or peripheral edema were present. Contrast-enhanced CT of the abdomen and pelvis revealed an 8.4 cm × 6.2 cm × 7.5 cm complex, multilobulated, fat containing left adrenal mass that also contained a more solid component with calcification that appeared to abut or possibly invade the pancreatic tail. This raised the suspicion for malignancy (Figures 1 and 2). There was also a 1.6 x 2 × 2.5 cm and 1.7 × 1.5 × 1.8 cm nodule in the right adrenal gland (Figures 1, 2). A CT of the abdomen and pelvis with and without contrast with a washout protocol was subsequently obtained. The macroscopic fat on the left adrenal mass had a Hounsfield units (HU) density of < -40. The soft tissue component with calcified portions had a precontrast density of 24 HU, a postcontrast density of 110 HU, and a delayed phase density of 86 HU. The absolute washout was 28%, and the relative washout was 22%. This is consistent with an indeterminant lesion and not adenoma. The left adrenal mass remained stable in size, and the suspected invasion of the pancreatic tail was again demonstrated. Pre, post, and delayed density characteristics of the right adrenal nodules were 29 HU precontrast, 104 HU postcontrast, and 50 HU on delayed images for the larger nodule in right adrenal and 33 HU, 139 HU, and 62 HU for the smaller right adrenal nodule. They both had an absolute and relative washout percentages of 72% and 52%, respectively, for the larger nodule and 73% and 55%, respectively, for the smaller right adrenal nodule. They were consistent with lipid-poor adenomas. A positron emission tomography (PET) scan revealed heterogeneous fluorodeoxyglucose (FDG) avidity localizing to the left adrenal mass. The nonfatty components in the left adrenal mass demonstrated a maximal standard uptake value (SUVmax) ranging from 5.6 to 6.3. The right adrenal gland, including the nodules, was also FDG avid with a maximal SUV of 5.8 (Figure 3). The fatty regions of the left adrenal mass were not metabolically active. The solid component in the left adrenal mass had an uptake on PET scan that was greater than the hepatic background. This can be seen with the adenomatous or hematopoietic components of myelolipoma but may also occur in malignant conditions. Benign adrenal lesions typically demonstrate SUV uptake values that are equivalent to or less than the hepatic background activity. Mildly avid foci in the mediastinum and right hilum were also identified, which were nonspecific and believed to represent subcentimeter reactive inflammatory nodes. There was no evidence for FDG avid nodal metastasis on the PET/CT. Staging CT of the chest was also negative for intrathoracic metastatic disease.

A complete biochemical laboratory evaluation was performed (Table 1). Blood tests revealed elevated aldosterone. However, plasma renin activity was normal (3.911 ng/mL/h), and serum potassium was 5.3 mmol/L, effectively ruling out primary aldosteronism. Plasma normetanephrine was borderline elevated; however, 24-hour urine total metanephrines were within normal limits, and the patient lacked any symptomatology characteristic of pheochromocytoma. Steroid precursors, dehydroepiandrosterone sulfate (DHEAS), and 17-hydroxyprogesterone were elevated, which raised the suspicion of malignancy. The constellation of this patient's clinical, laboratory, and radiographic features, namely, short stature with concomitant elevation in ACTH and steroid precursors, dehydroepiandrosterone sulfate (DHEAS), and 17-hydroxyprogesterone, in addition to bilateral adrenal lesions, also raised the suspicion of CAH due to 21-OHD. Thyroid function tests were consistent with subclinical hypothyroidism (elevated TSH, with normal free T4), which was treated; thyroid peroxidase antibodies were very high, consistent with Hashimoto's thyroiditis. Baseline cortisol level was low, and ACTH was markedly elevated at 160 pg/mL.

The decision was made to proceed with surgical resection of the left adrenal gland secondary to inability to exclude adrenal malignancy due to large size, apparent invasion into the tail of the pancreas, indeterminate (nonadenoma) washout characteristics, and FDG avidity. The patient underwent exploratory laparotomy with left adrenalectomy and distal pancreatectomy. Hydrocortisone 50 mg IV was given preoperatively and continued every 6 hours in the perioperative period. The left adrenal mass extended high into the retroperitoneum, abutting but not involving the splenic hilum and upper pole of the left kidney, with fibrinous attachments to the tail of the pancreas anteriorly. There was no lymphadenopathy or other signs of metastatic disease present on exploration. Frozen section of the adrenal mass was positive for myelolipoma. Final pathology documented a 10.0 cm × 8.2 cm × 6.5 cm adrenal gland weighing 175 g, with the left adrenal mass positive for AML, and one adrenal cortical adenoma. The tail of the pancreas contained one microscopic, well differentiated 2 mm neuroendocrine tumor, with a Ki67 labeling index of <3% and a low mitotic rate.

The patient was continued on hydrocortisone and levothyroxine postoperatively and discharged home on postoperative day 5 after an uneventful hospital course. Given the significant elevation of the serum 17-hydroxyprogesterone level, his short stature was reportedly lower than his biological paternal and maternal heights (72 inches and 64 inches, respectively), and on the presence of myelolipoma on final pathology, the diagnosis of CAH due to 21-OHD was highly suspected. Full gene analysis sequencing for the CYP21A2 gene was performed to evaluate for suspected CAH due to 21-OHD. The results revealed that both copies of the CYP21A2 gene carry the pathogenic variant c.293–13C > *G*, supporting diagnosis of CAH due to 21-OHD. Testicular ultrasound was also performed to rule out adrenal rest tumors. No adrenal rest tumors were seen, although testicular size was somewhat small bilaterally, with maximum dimension of the right and left testicle at 3.0 cm. Hydrocortisone therapy will be continued indefinitely to potentially prevent further growth of the remaining contralateral adrenal gland. The patient's plasma renin activity was somewhat elevated when measured following hospital discharge, and fludrocortisone 0.1 mg daily was added to the hydrocortisone 10 mg twice daily dose.

## 3. Discussion

AML was initially most commonly discovered on autopsy; however, as diagnostic imaging has become more sophisticated, it is most commonly diagnosed incidentally on imaging studies obtained for other indications [1]. Although the exact pathophysiological mechanism in tumor formation remains unclear, elevated ACTH levels and persistent stimulation by ACTH, as in both CAH and Cushing's disease, have been implicated to play a role in the development of AML [7–11]. In fact, in patients with untreated CAH, the incidence of both adrenal hyperplasia and adrenal neoplasms increases with increased severity of the enzymatic defect, especially in myelolipomas occurring within an adenoma [8, 9]. In a systematic review of 420 cases of AML, 10% of cases were associated with CAH [12]. Similarly, Falhannar et al. reported that 45% of genetically proven CAH carriers had adrenal incidentalomas, and of these, greater than 50% had bilateral adrenal lesions [13].

CAH is an autosomal recessive disorder that is most commonly due to 21-OHD due to mutation in the CYP21A2 gene [10, 14, 15]. Less commonly, CAH may occur secondary to 17-hydroxylase deficiency and 11-hydroxylase deficiency [11]. 21-OHD, in turn, results in impaired production of cortisol and elevated adrenocorticotropic hormone (ACTH), which enhances adrenal androgen secretion. Increased stimulation by ACTH is considered the pathophysiological mechanism behind adrenal hyperplasia, but whether ACTH hypersecretion also causes tumor growth is unclear [11]. Many patients with CAH who are diagnosed with AMLs are either untreated or their condition is poorly controlled with chronic elevations in ACTH [11]. CAH due to 21-OHD has been categorized as classical and nonclassical forms. The classical form is traditionally thought to manifest as the simple virilizing or salt wasting forms. However, in contemporary teaching, the distinction between purely “salt wasting” and purely “simple virilizing” subtypes of classical CAH should be discouraged [16], and CYP21A2 allelic variants and their phenotypic manifestations are believed to occur on a continuum. In nonclassical 21-OHD, in which there is a partial hydroxylase enzyme deficiency, 20–50% of normal enzyme function is present, sufficient to adequately suppress ACTH over secretion [15]. In patients with milder enzymatic defects or with nonclassic, attenuated form of 21-OHD, postnatal virilization, menstrual irregularities, and short stature are sometimes present [7]. At birth, patients with nonclassical adrenal hyperplasia have normal genitalia with normal baseline 17-OH progesterone levels. Growth may eventually be arrested in these patients due to early epiphyseal fusion compromising final height [15]. Suchartlikitwong et al. presented a case of a 39-year-old male with untreated CAH presenting with acute adrenal insufficiency and severe virilization as noted by short stature, skin hyperpigmentation, micropenis, and no palpable testes, who was found to have bilateral AMLs [17]. Overall, the frequency of germ cell mutation in CYP21A2 was 21% in patients with bilateral incidentalomas [11].

All incidentally diagnosed adrenal masses should undergo hormonal evaluation to determine the presence of a functional tumor. It is estimated that 10–20% of AMLs have been associated with some endocrine disorders, 85% of which involve the pituitary-adrenal axis, including Cushing syndrome, congenital adrenal hypoplasia with 21-OHD or 17-hydroxylase deficiency, Conn syndrome, and androgenital syndrome due to primary aldosteronism [6, 7, 18]. AMLs have also been associated with increased catecholamine production and pheochromocytoma [18, 19]. In some cases, the endocrine disorder is due to myelolipomatous metaplasia within a functioning cortical neoplasm, most commonly adrenocortical adenoma; however, there have been cases reported in which myelolipoma itself has been associated with hyperfunctioning of the adrenal cortex without a recognizable adrenocortical lesion [7]. Thus, myelolipomatous adrenal lesions, whether true AMLs or adrenocortical neoplasms with myelolipomatous elements, may lead to hyperfunction of either the adrenal cortex or the adrenal medulla [6, 7, 18, 19].

The key diagnostic feature of AML is the presence of adipose tissue within an adrenal mass. However, in some instances, as in our patient, diagnosing AML based on radiological imaging features is not straightforward, especially as AMLs may occur synchronously with other benign or malignant adrenal lesions [2, 6]. In addition, adrenal tumors may be of uncertain malignant potential when tumor characteristics lack major criteria for malignancy such as high mitotic activity, atypical mitosis, or vascular invasion, but demonstrate minor criteria, such as large size, tumor necrosis, capsular invasion, or moderate FDG avidity on PET scan [20, 21]. In the presence of bilateral disease, presence of systemic disease, including metastatic neoplasms, should be considered [5]. Differential diagnoses and suspected preoperative diagnoses of liposarcoma, retroperitoneal sarcoma, adrenal metastatic lesion, and adrenocortical carcinoma have been reported in the literature for adrenal masses later proven to be benign AMLs [2, 5, 22]. AMLs with hemorrhage and necrosis, especially when occurring within an adrenal adenoma, as reported by Al-Brahim et al. may erroneously suggest a diagnosis of adrenocortical carcinoma on radiologic imaging [2]. Lastly, AML may be associated with a false positive 18F-FDG PET scan due to possible FDG uptake related to the trophic adrenal effect of ACTH [1, 23], as in our case which demonstrated 18-FDG avidity. Similarly, Kocak et al. reported a case of a 46-year-old male with benign, bilateral AML secondary to CAH confirmed on pathology following bilateral adrenalectomy, which demonstrated unilateral hypermetabolic activity of the right mass on preoperative PET scan with a maximum SUV of 11.8 [24]. Malignant transformation or metastatic spread of AML has not been reported.

The presence of macroscopic fat tissue in an adrenal lesion does not automatically rule out the possibility of a malignant lesion, which was another reason why we ultimately sent our patient to surgery. It is imperative for clinicians to be mindful of this when evaluating patients with this important radiographic feature. Macroscopic fat in an adrenal lesion has been described in adrenocortical carcinomas, adrenal oncocytic neoplasms, which are very rare adrenal neoplasms that can sometimes behave in a malignant fashion, and collision tumors, which are tumors composed of two different adjacent histologies, such as myelolipoma with coexisting adrenocortical carcinoma or myelolipoma with a metastatic lesion from a separate extra-adrenal primary malignancy [25–28].

Management of AML is dependent on size of the lesion, clinical presentation, and inability to exclude cancer. Surgery is typically reserved for lesions that are symptomatic, large (>6-7 cm), increasing in size on serial imaging, or with indeterminant malignant potential [19, 24, 29, 30]. While AMLs are most commonly clinically quiescent and diagnosed incidentally, rarely they come to clinical attention due to symptomatology. Abdominal and/or flank pain due to mass effect or tumor necrosis, vomiting, hematuria, rupture, and life-threatening hemorrhage have been described in the literature [6]. In the case of bilateral AML, which most frequently occurs in individuals with CAH, monolateral symptoms are the most commonly reported clinical feature. Monolateral adrenalectomy is recommended for the symptomatic tumor, leaving the contralateral adrenal gland in place in order to preserve some adrenal hormone function. Bilateral adrenalectomy is reserved for symptomatic tumors or tumors >7 cm. With proper medical management, the remaining AML will hopefully remain stable (though this is not guaranteed), as in the case reported by Zattoni et al., in which a patient with bilateral AML (17 cm × 12 cm (−20 HU) on the right and 2.3 cm × 2.5 cm (−5 HU) on the left) underwent an open right adrenalectomy with radiological surveillance demonstrating no recurrence on the right side and a left lesion that remained stable over time [30]. The presence of bilateral adrenal lesions should raise the suspicion for a systemic condition, including infection or metastasis from an extra-adrenal primary tumor, as well as conditions of ACTH excess, including CAH or a hereditary pheochromocytoma syndrome [5]. In cases where the diagnosis of adrenal malignancy is uncertain, particularly in instances where the adrenal incidentaloma demonstrates imaging characteristics that are suspicious for malignancy and/or the patient has a known primary malignancy, there may be a diagnostic role for percutaneous fine needle aspiration of the mass; however, this carries an associated potential risk of bleeding, tumor rupture, and subsequent tumor dissemination [31].

In terms of our case, based on CT washout data, the right adrenal lesion was most consistent with benign, lipid-poor adenoma. The larger left adrenal lesion was probably myelolipoma, but a superimposed malignancy could not be ruled out based on the foci of FDG avidity, greater than the hepatic background, within the solid component of the left adrenal mass. Our patient underwent surgical resection due to the large size of the left adrenal lesion and an overall clinical picture that was highly suspicious for a malignant process, including radiographic evidence of possible involvement of the pancreatic tail. He was subsequently diagnosed with AML in association with CAH, classic type, secondary to 21-OHD. The patient is doing well and remains on steroid replacement therapy consisting of hydroxycortisone 10 mg twice daily and fludrocortisone 0.1 mg once daily in addition to levothyroxine 50 mcg daily for treatment of subclinical hypothyroidism. Although our patient did not have overt aldosterone deficiency, fludrocortisone 0.1 mg daily, in addition to hydrocortisone 10 mg twice daily, dose steroid therapy was initiated, in compliance with the current recommendations that suggest that most nonhypertensive adults with classic CAH benefit from continued fludrocortisone treatment. Also, in patients with 21-hydroxylase deficiency-induced CAH, mineralocorticoid replacement may permit dose reduction of daily glucocorticoid requirements, which in turn may reduce the risk of iatrogenic Cushing syndrome due to overreplacement of glucocorticoids [16, 32]. Depending on subsequent androstenedione and testosterone levels, with a goal ratio in CAH of <2 (normal is < 0.5), the hydrocortisone dose can be adjusted [32]. The initial dose of hydrocortisone given to the patient on discharge after surgery was 10 mg thrice daily, but this was reduced to 10 mg twice daily when outpatient labs revealed a testosterone level of 73.6 ng/dL and a serum androstenedione level of 17 ng/dL (normal: 40–150 ng/dL). It is possible that steroid therapy will help prevent further growth of the remaining adrenal gland, obviating the need for a future right adrenalectomy. Although our patient will be maintained on hydrocortisone and fludrocortisone indefinitely, had his remaining adrenal gland been resected, he would be glucocorticoid and mineralocorticoid dependent with daily steroid replacement required for survival, a far more potentially dangerous clinical scenario than he is currently in. Regularly scheduled serial clinical monitoring and imaging surveillance to assess for tumor growth will be conducted. Periodic monitoring for adrenal rest tumors via testicular ultrasound will also be performed.

## 4. Conclusion

AML may represent a diagnostic dilemma for surgeons and endocrinologists, particularly when occurring synchronously with other benign or malignant adrenal lesions, lesions in nearby structures, and in lesions with associated tumor rupture or necrosis, which may give a radiographic appearance of heterogeneity, suggesting a radiologic diagnosis of adrenocortical carcinoma. This is especially true for AMLs occurring within an adrenal adenoma, in which there is a radiologic appearance of a suspicious heterogeneous lesion with a rim of normal appearing adrenal tissue. In addition, large size and FDG avidity on PET scan may occur in AML, which may also resemble a malignant lesion. Surgeons should be aware of these findings associated with AMLs to avoid diagnostic pitfalls which may result in over diagnosing malignancy and over treating a benign disease.

## Figures and Tables

**Table 1:** Endocrinologic laboratory tests.

Lab test	Patient value	Reference range
Gastrin	177 pg/mL	<100 pg/mL
Renin activity, Pl	6.683 ng/mL/h	0.167–5.380 ng/mL/h
Aldosterone	41.8 ng/dL	</ = 23.2 ng/dL
Plasma normetanephrine, Pl	147.6 pg/mL	0.0–125.8 pg/mL
Plasma metanephrine, Pl	25.2 pg/mL	0.0–88.0 pg/mL
Cortisol AM^*∗*^	0.7 ug/dL	6.0–18.4 ug/dL
Cortisol AM	4.6 ug/dL	6.0–18.4 ug/dL
DHEAS^*∗*^	299 ug/dL	95.0–530.0 ug/dL
DHEAS	598 ug/dL	95.0–530.0 ug/dL
24 hours urine metanephrine	63 mcg/24 h	44–261 mcg/24 h
24 hours urine normetanephrine	169 mcg/24 h	119–451 mcg/24 h
24 hours total urine metanephrine	232 mcg/24 h	211–646 mcg/24 h
Fractionated 24-hour urine epinephrine	4 ug/24 h	0–20 ug/24 h
Fractionated 24-hour urine norepinephrine	23 ug/24 h	0–135 ug/24 h
Fractionated 24-hour dopamine	276 ug/24 h	0–510 ug/24 h
Testosterone	447.0 ng/dL	249.0–836.0 ng/dL
17-Hydroxyprogesterone	6078 ng/dL	27–199 ng/dL
11-Heoxycortisol	40 ng/dL	</ = 76 ng/dL
TSH	7.85 uIU/mL	0.27–4.20 uIU/mL
Free T4	0.9 ng/dL	0.9–1.8 ng/dL
Thyroid peroxidase antibody	1608.0 IU/mL	</ = 34.9 IU/mL
Thyroglobulin antibodies	<20.0	</ = 40.0 IU/mL
ACTH	160.0 pg/mL	7.2–63.3 pg/mL
21-Hydroxylase antibodies	Negative	Negative

^*∗*^Status post 1 mg dexamethasone suppression test.

## Abbreviations

AML: Adrenal myelolipoma
CAH: Congenital adrenal hyperplasia
CT: Computed tomography
FDG: Fluorodeoxyglucose
HU: Hounsfield units
PET: Positron emission tomography
SUV: Standard uptake value
21-OHD: 21-Hydroxylase deficiency.
